# Genome-Wide Association Study of Grape Texture Based on Puncture

**DOI:** 10.3390/ijms252313065

**Published:** 2024-12-05

**Authors:** Meiling Lin, Lei Sun, Xuewei Liu, Xiucai Fan, Ying Zhang, Jianfu Jiang, Chonghuai Liu

**Affiliations:** 1 Zhengzhou Fruit Research Institute, Chinese Academy of Agricultural Sciences, Zhengzhou 450000, China; lml18831276234@163.com (M.L.); sunlei01@caas.cn (L.S.); 15738246253@163.com (X.L.); fanxiucai@caas.cn (X.F.); zhangying05@caas.cn (Y.Z.); 2 ZhongYuan Research Center, Chinese Academy of Agricultural Sciences, Xinxiang 453424, China

**Keywords:** grape, texture, GWAS, puncture method, candidate genes

## Abstract

Grapes are grown extensively around the world and play a crucial role in overall fruit production globally. The quality of the grape is largely determined by the texture of the flesh, making it a key focus for grape breeders. Our study was conducted on 437 grape accessions using a puncture method to analyze berry texture characteristics. The results reveal strong correlations among the five texture parameters of grape accessions. Following the GWAS analysis using 2,124,668 population SNPs, 369 significant SNP locations linked to the grape berry texture were discovered. Through the process of gene annotation and expression analysis in the localization regions, several genes potentially linked to berry texture were identified, including *E13A*, *FIS1A*, *CML35*, *AGL2*, and *AGL62*. *E13A*, *FIS1A*, and *CML35* were identified as potentially more relevant to grape berry texture based on gene expression analysis. Further investigation through transient transformation demonstrated that overexpressing *E13A* and *CML35* resulted in notable changes in grape pulp texture. During this study, the berry textures of 437 grape accessions were comprehensively evaluated, and several important candidate genes were screened based on GWAS and analysis of gene function. This discovery paves the way for future research and breeding initiatives related to grape berry texture.

## 1. Introduction 

Grape is one of the most widely cultivated tree species in the world, and plays a key role in the global fruit production [1]. As an economically significant fruit, it is extensively cultivated for various applications such as fresh consumption, wine production, and juice extraction [2,3]. It has a high nutritional value and is beneficial for health [4,5]. During the storage process of grapes, a large amount of internal tissue moisture loss, flesh softening, and poor flavor affect the marketability and storability of berries, seriously restricting the development of their industrial chain [6]. 

The texture of grape berries can generally be used to measure their freshness. Berry texture is a key parameter of fruits, which not only affects consumers’ acceptance of fruits, but also has a significant impact on the transportation, storage, and commercial value of fruits in the market after harvesting [7,8]. The flesh texture is one of the important indicators for the commercialization of grapes. A crunchy texture is more popular with consumers, so crunchy flesh table grapes have also become one of the important breeding objectives of grape breeders. In grape breeding programs, identifying crunchy varieties is of paramount importance, as crosses between non-crunchy fleshed varieties seldom yield offspring with crunchy characteristics. Within the cultivation of table grapes, crunchy-textured varieties serve as invaluable genetic resources [9]. Peel hardness is one of the important factors affecting the overall texture characteristics of grapes. Higher peel toughness and hardness can reduce the damage to berries during harvesting, transportation, and processing, and maintain their integrity [10]. At the same time, it also helps to maintain the internal moisture of berries, prevent excessive loss of moisture, and reduce the rate of berry cracking [11]. Grapes, as perennial woody plants, share with other fruit trees the traits of extended juvenile phases and high genetic diversity. Traditional breeding methods typically require 4 to 5 years for these plants to mature and begin fruiting [12,13]. The process is time-consuming, labor-intensive, and requires significant investment. However, with advances in modern molecular biology, botanists have turned to molecular breeding techniques. These methods enable the pre-selection of a vast array of individuals and the targeted enhancement of genetic regions, facilitating the development of improved varieties and the achievement of desired phenotypes [14]. Molecular breeding offers the potential to significantly reduce the duration of the breeding cycle, expedite the breeding process, and enhance the overall breeding efficiency [1]. 

The process of fruit hardness reduction is related to changes in the cell wall, including the degradation of cell wall components caused by changes in cell wall metabolism and changes in enzyme activity related to cell wall degradation. Enzymes related to cell wall modification may play a role in fruit softening, including polygalacturonase (PG), pectin methylesterase (PME), β-galactosidase (β-gal), cellulase, and xyloglucan transglycosylase (XTH). PG is one of the pectinases most involved in fruit softening research. So far, the efficacy of PG in promoting fruit softening and reducing hardness has been confirmed in tomatoes [15], apples [16], and figs [17]. XTH can reshape xyloglucan, which is the main hemicellulose in dicotyledonous plants. It has been proven that there is a strong correlation between the metabolism of xyloglucan and fruit hardness [18]. According to reports, the high ripening-related gene *DKXTH8* in persimmon fruit promotes fruit ripening and decreases fruit firmness [19]. Pectinesterase plays an important role in cell wall metabolism. Silencing *PMEU1* will accelerate the softening process and reduce the firmness of the tomato during ripening [20]. With the *SIEXP1* gene’s expression during fruit ripening being closely associated with fruit softening, analysis of tomato mutants has revealed that a deficiency in *SIEXP1* function results in increased fruit firmness and a delay in ripening [21]. β- galactosidase is a cell wall hydrolase. In strawberries [22] and peaches [23], the downregulation of β- galactosidase will delay fruit softening. In peaches, the fruit firmness decreased with the decrease of *PPIAA1/9* expression [24]. In addition, some plant hormones can also affect fruit softening by affecting the activity of cell wall-degrading enzymes. Silencing the acnced1 gene in kiwi fruit has been shown to inhibit the synthesis of abscisic acid (ABA) and maintain fruit firmness [25]. Ethylene treatment at the later stage of pear development increased the expression of genes related to cell wall degradation and promoted fruit softening [21]. Brassinosteroids (BR) are a type of polyhydroxysteroid plant hormone [22]. The treatment of peaches with BR could inhibit the activity of pectin-degrading enzymes and delay the decline in fruit firmness [23]. In addition, the softening of fleshy fruits is also related to changes in transpiration water loss and turgor pressure [26]. In grapes, *VvPL15* was identified through bioinformatics analysis as a key gene for predicting pectin lyase and related to the ripening and softening processes of grape berries [24]. 

However, in comparison to fruits from apple, peach, and other fruit trees, research on the texture of grape berries is comparatively limited. In this study, the five texture traits of grapes were measured, and the phenotypic data obtained were combined with the previous re-sequencing data of our research group for genome-wide association analysis. The analysis aims to identify gene loci and candidate functional genes related to grape texture.

## 2. Results

### 2.1. Phenotypic Analysis of Berry Texture 

**Figure 1:**
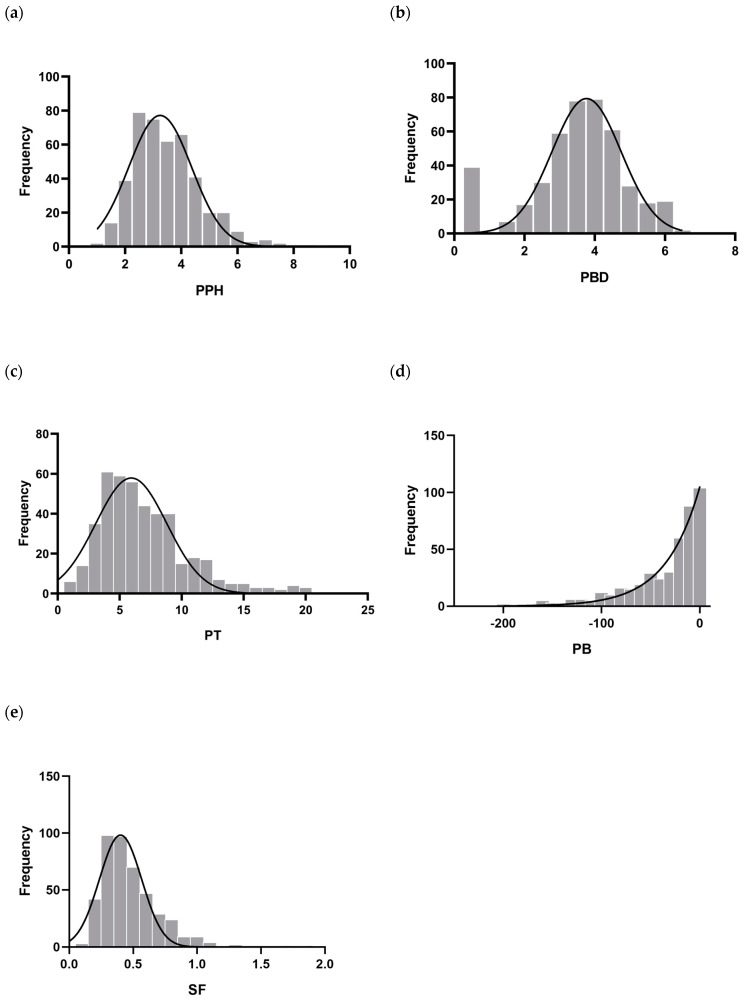
Frequency distribution of (**a**) PPH: Pericarp hardness; (**b**) PBD: Pericarp break distance; (**c**) PT: Peel toughness; (**d**) PB: Pulp brittleness; (**e**) SF: Sarcocarp firmness.

In this study, a natural population comprising 437 grape accessions, including table and wine types, was selected for analysis (Table S1). Five key textural characteristics of grapes were measured and statistically evaluated. The pericarp hardness (N) of 437 grape accessions ranged from 1.173 to 9.089 N, and with an average of 3.488 N, the pericarp hardness of “Cuihong” and “Pensal blanco” was the highest. The peel rupture distance (mm) was the downward displacement of the probe from contacting the peel to puncturing the peel. The peel rupture distance ranged from 0.031 to 6.415 mm, with an average of 3.496 mm. The grapes with large peel rupture distances were all table grapes, and the largest was the Muscat violet common. Peel toughness (N.s) is the product of the force value of the first peak of the puncture curve and the running distance. The peel toughness of all tested materials ranged from 0.859 to 19.813 N.s, and the difference was nearly 23 times. The flesh brittleness of different types of grape accessions varied greatly, ranging from 0.482 to 332.684 N/s, with a size difference of nearly 690 times. Table grapes had the most brittle flesh, and the “Cuihong” accession was the most brittle. Pulp firmness refers to the average firmness of grape berry pulp. The flesh hardness of all tested materials ranged from 0.091 to 1.843, with an average of 0.476 N. The flesh hardness of Eurasian species was higher than that of European and American hybrids. The coefficient of variation (CV) values indicated a wide range of variability among the traits. Peel hardness showed the lowest CV (0.342), while pulp brittleness had the highest CV, reaching 1.185 (Table 1). Based on the phenotypic data from the 437 grape accessions, the distribution of the five textural traits was examined. Except for pulp brittleness, which showed a clear continuous distribution, the other traits approximated a normal distribution (Figure 1).

### 2.2. Correlation Analysis of Berry Texture Traits

**Figure 2:**
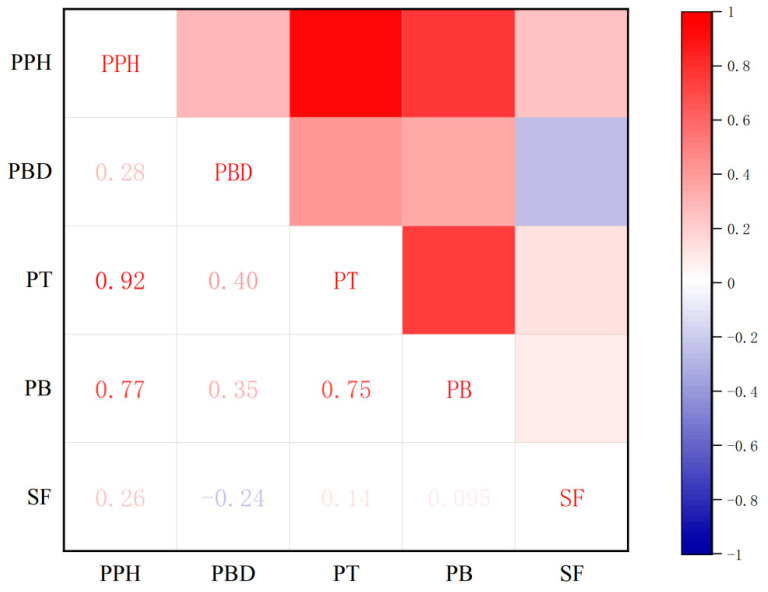
Phenotypic correlation analysis of five texture traits. Color depth indicates the intensity of correlation coefficient, red indicates positive correlation, and blue indicates negative correlation. The *p* value of significant correlation is shown at the bottom. PPH: Pericarp hardness; PBD: Pericarp break distance; PT: Peel toughness; PB: Pulp brittleness; SF: Sarcocarp firmness.

The results indicate that the pericarp hardness is highly and positively correlated with the peel toughness and the pulp brittleness, with correlation coefficients of 0.92 and 0.77, respectively. This suggests that an increase in the pericarp hardness of the grape is paralleled by an increase in peel toughness, and the greater the pericarp hardness, the greater the decrease in biting force and the greater the brittleness when biting into the grape. The distance at which the pericarp breaks is positively correlated with peel toughness and flesh crispness, indicating that, the harder the skin, the greater the pulp brittleness and the greater the displacement of the probe when punctured by a texture analyzer. The crispness of the flesh is significantly and positively correlated with peel toughness (0.75), but has a low correlation with sarcocarp firmness, indicating that, the greater the pulp brittleness, the lower the pericarp hardness (Figure 2).

The analysis of the grape accession revealed that the majority of the five measured textural parameters are highly or extremely significantly correlated, underscoring the multifaceted nature of grape berry texture as an aggregate of various interrelated traits. 

### 2.3. Cluster Analysis Based on Berry Texture Traits

**Figure 3:**
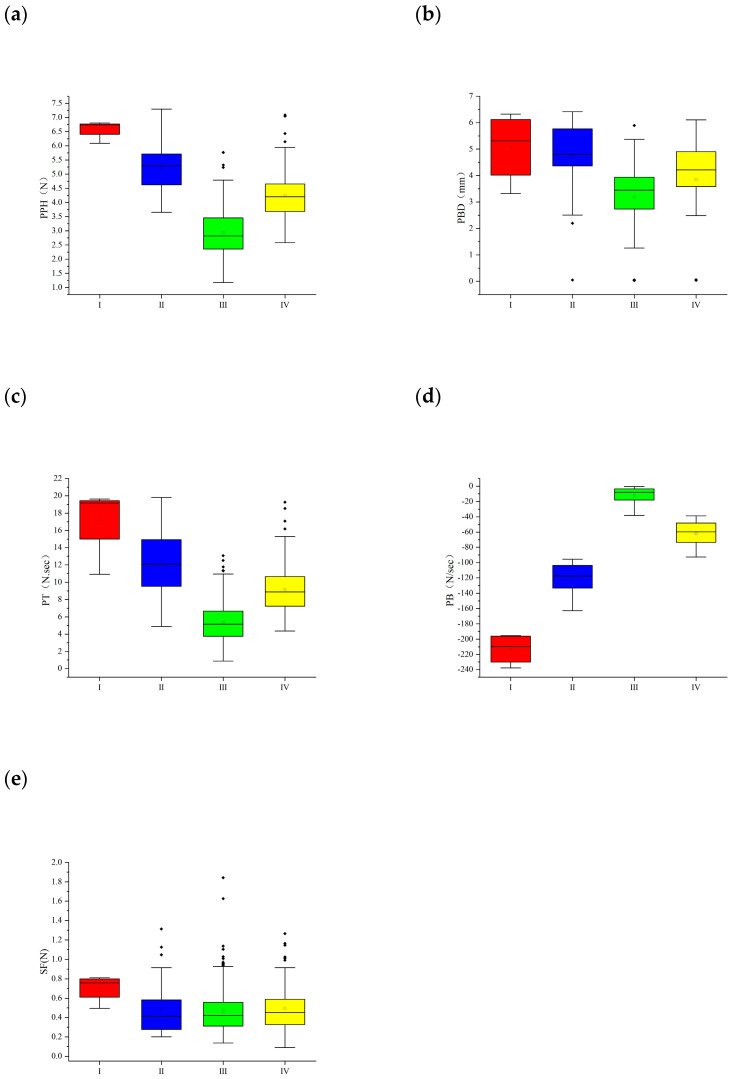
Box chart showing the average, median, and range of phenotypes of four grape groups, (**a**) PPH: Pericarp hardness; (**b**) PBD: Pericarp break distance; (**c**) PT: Peel toughness; (**d**) PB: Pulp brittleness; (**e**) SF: Sarcocarp firmness. The “.” in the figure represents an outlier.

Based on the analysis of five texture traits, grapes were divided into four groups (I, II, III, IV). The first group included 41 accessions such as “Golden Muscat”. The peel hardness, peel toughness, and peel break distance of this group were large, and the proportion of Eurasian species in this group was large (Figure 3). The second category included 106 accessions such as “Crimson Seedless” and “Gold”, most of which were table accessions. Group III contained 57 accessions. The five texture traits in groups II and III were in the middle position. Group IV contained 233 accessions, of which the peel hardness, pulp break distance, and pulp brittleness were low. 

**Figure 4:**
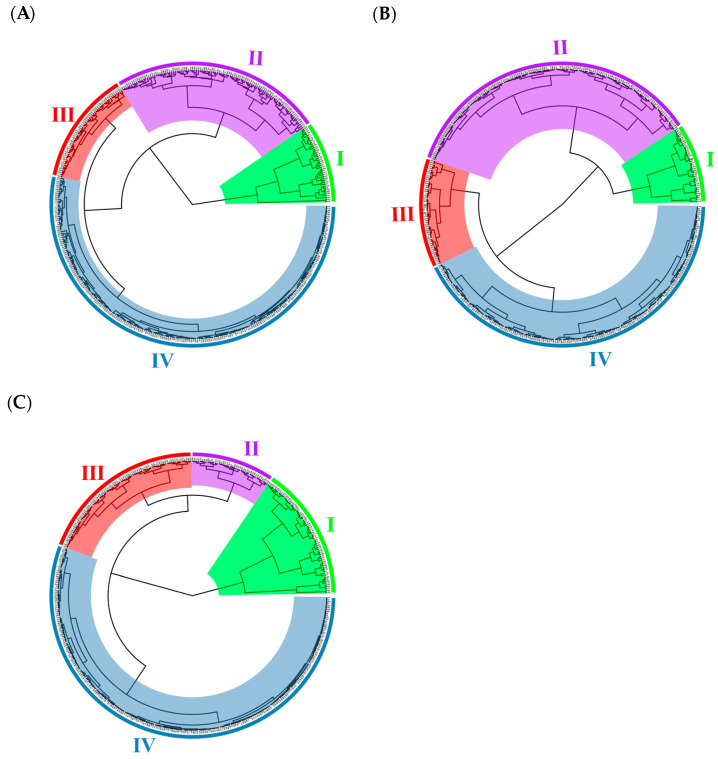
Cluster analysis of texture traits of 437 grape accessions. (**A**) Comprehensive clustering of five texture traits; (**B**) Pericarp hardness; (**C**) Pulp brittleness.

The tested accessions were also divided into four groups (I, II, III, IV) by cluster analysis using single phenotypic traits. The clustering results of flesh brittleness were the closest to the comprehensive clustering results of five texture traits, and the clustering results of 362 accessions were the same (Figure 4B). The cluster results of pericarp hardness were second, and 235 accessions had the same results (Figure 4C). The cluster results of peel toughness and flesh hardness were the most different from the overall cluster results, and only 130 accessions were the same (Figure S1). The results show that flesh brittleness and peel hardness contributed the most to the classification in the cluster, which could be used as the basis for the classification of berry texture and could represent the overall berry texture.

### 2.4. GWAS of Berry Texture

**Figure 5:**
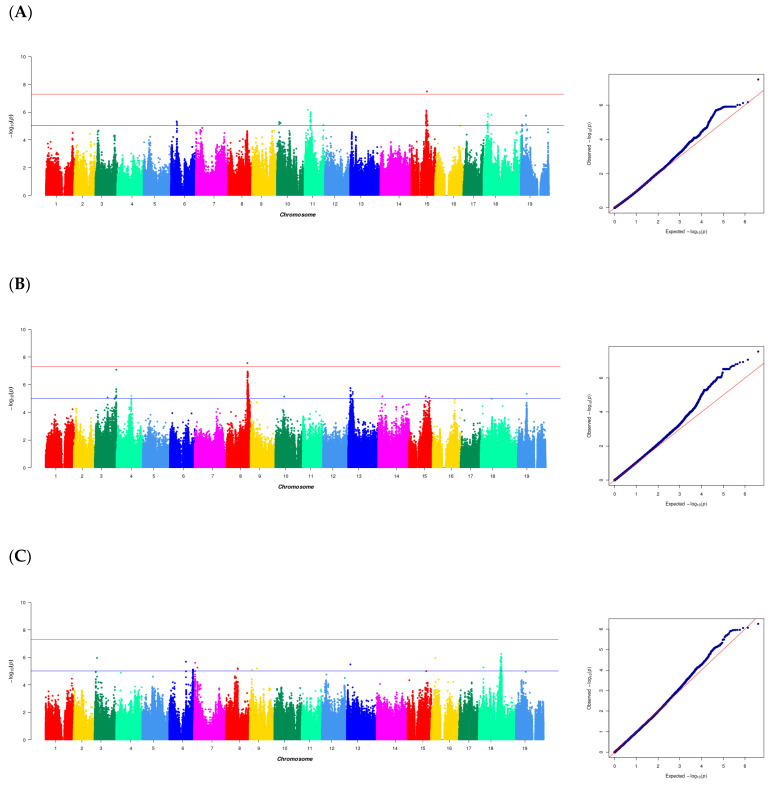
Genome-wide association analysis (GWAS) Manhattan plot and Q-Q plot of (**A**) PBD: Pericarp break distance; (**B**) Pulp brittleness; (**C**) Sarcocarp firmness. The red horizontal line indicates the significance threshold (−Lg *p* > 7). The blue horizontal line indicates the significance threshold (−Lg *p* >5).

Using the PNT2T grape genome as a reference genome [27], a stable population was established by combining 437 grape accessions. Through quality control and screening of the original data, a dataset including 2,124,668 SNP variants was established. The relationship between five berry textures and SNPs of 437 grape accessions was analyzed with the MLM model. A total of 369 SNPs were identified by GWAS using the MLM model, which were distributed on all grape chromosomes except staining No. 1, 2, 5, 12, and 17, and were significantly correlated with five berry texture traits (Table S2). Among them, 13 SNP markers were significantly correlated with peel hardness, and 4 SNP markers were significantly correlated with peel toughness. However, the two traits were excluded from further analysis because the *p* value was not significant (Supplementary Figure S2). A total of 94 significant markers associated with peel break distance were located on seven chromosomes, and these SNPs could explain 4.3–6.7% of the phenotypic variation in peel break distance (Figure 5A). Among them, only the significant SNP on chromosome 11 was associated with the peel rupture distance. Two SNP peaks, 11_3126218 and 15_15166809, were found on chromosomes 11 and 15. A total of 199 SNP loci were found to be significantly associated with pulp brittleness, distributed on seven chromosomes. The phenotypic variation (PVE) explained by these SNPs ranged from 4.3% to 6.8%. Only the significant SNP on chromosome 14 was associated with pulp brittleness. It is worth noting that the SNP site 8_20379794 on chromosome 8 is prominent in the Manhattan curve. A total of 58 potential SNP loci associated with this trait were identified, and these SNPs were distributed on six chromosomes and accounted for 4.3–5.5% of the phenotypic variation in sarcocarp firmness. The significant SNPs on chromosomes 7 and 16 were only related to sarcocarp firmness. The most significant markers were 18_22038237 and 18_21756428 on chromosome 18.

### 2.5. Candidate Gene Screening

Based on the SNP markers significantly associated with berry texture traits in the grape reference genome, candidate genes were screened near the significant SNP loci. A total of 155 genes were identified near 369 significant SNPs (Table S3). Using the Ensembl Plants Database (http://plants.ensembl.org/index.html/ accessed on 1 November 2023) and the National Biotechnology Information Center (NCBI) database (https://www.ncbi.nlm.nih.gov/ accessed on 1 November 2023), the candidate genes were annotated and predicted. A total of 12 genes were preliminarily screened out through gene annotation. These genes include receptor kinases, transferases, glycosidases, oxidoreductases, transfer proteins, binding proteins, etc. (Table 2).

On chromosome 11, 11_3126218, which is related to the distance of pericarp rupture, and genes near the locus are annotated as related to Protein Mpv17 and t RNA(Ile)-lysidine synthase. On chromosome 15, the gene near this peak is annotated as being related to nitrate regulatory gene 2 protein and leucine carboxy methyltransferase 1 homolog. In addition, genes near the 18_84130876 locus on chromosome 18 and three surrounding SNP loci are annotated as glucan 1,3-beta-glucosidase. For pulp brittleness, 8_20379794 is highlighted in the Manhattan map, and the surrounding genes are annotated as nonsense-mediated mRNA decay factor SMG7. The gene near 8_20671485 is annotated as mitochondrial fission 1 protein A, which plays a crucial role in mitochondrial dynamics. Another gene, *AGL62*, located near the 8_21018177 locus, is annotated as an agave-like MADS-box protein AGL62 related to flower development. Possible calcium binding protein CML35 is annotated on chromosome 11. In addition, genes near two loci 8_20244131 and 8_20250484 are associated with *AGL2*. These genes are annotated as α-glucosidase 2 and identified as homologous genes of AGL9, indicating their conservative role during development. On chromosome 18, the gene at SNP site 18_22038237, which is associated with meat hardness, is annotated as an uncharacterized oxidoreductase At4g09670. The gene near site 8_11881442 on chromosome 8 is non-specific lipid transfer protein P5, which may play a role in wax or keratin deposition in the cell walls of expanded epidermal cells and some secretory tissues.

### 2.6. Expression of Candidate Genes During Berry Development Stage

**Figure 6:**
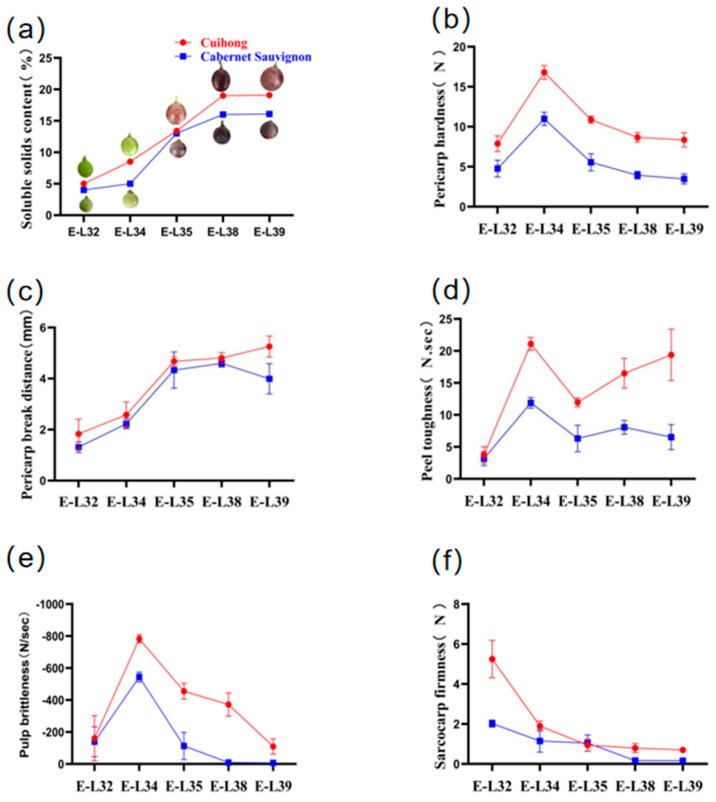
Physiological and biochemical indices during fruit ripening of “Cuihong” and “Cabernet Sauvignon” grapes at different developmental stages. (**a**) Soluble solids content (SSC); (**b**) Pericarp hardness; (**c**) Pericarp break distance; (**d**) Peel toughness; (**e**) Pulp brittleness; (**f**) Sarcocarp firmness. “Cabernet Sauvignon” is represented by blue squares; “Cuihong” is represented by red circles. The five nodes of the coordinate axis are E-L 32, E-L 34, E-L 35, E-L 38, and E-L 39.

During the fruit development process, the soluble solids content (SSC) of both Cabernet Sauvignon and “Cuihong” exhibited a continuous upward trend. Overall, the SSC of “Cuihong” remained higher than that of “Cabernet Sauvignon” throughout the process. A rapid increase in SSC was observed during the Veraison stage (E-L 35), and by the ripening stage (E-L 38) and the full ripe stage (E-L 39), the SSC of the berries had essentially stabilized, with “Cuihong” approaching 19.2% and “Cabernet Sauvignon” stabilizing at around 15%. From the bunch closure stage (E-L 32) to the softening stage (E-L 34), there was no significant change in berry weight, but during the Veraison stage (E-L 35), the berries began to enlarge, with a significant increase in individual fruit weight, essentially reaching their inherent weight. The changes in skin hardness and flesh crispness of both “Cabernet Sauvignon” and “Cuihong” were consistent, showing an initial increase followed by a decrease, with a rapid increase to the maximum value during the softening stage (E-L 34). However, throughout the entire fruit development process, the peel hardness and pulp brittleness of “Cuihong” were significantly higher than those of “Cabernet Sauvignon”. The sarcocarp firmness continuously decreased throughout the development period, with the berries rapidly softening during the softening stage, the trend of changes in the Pericarp break distance is opposite, yet the textural parameters of “Cuihong” remained consistently higher than those of “Cabernet Sauvignon” (Figure 6).

**Figure 7:**
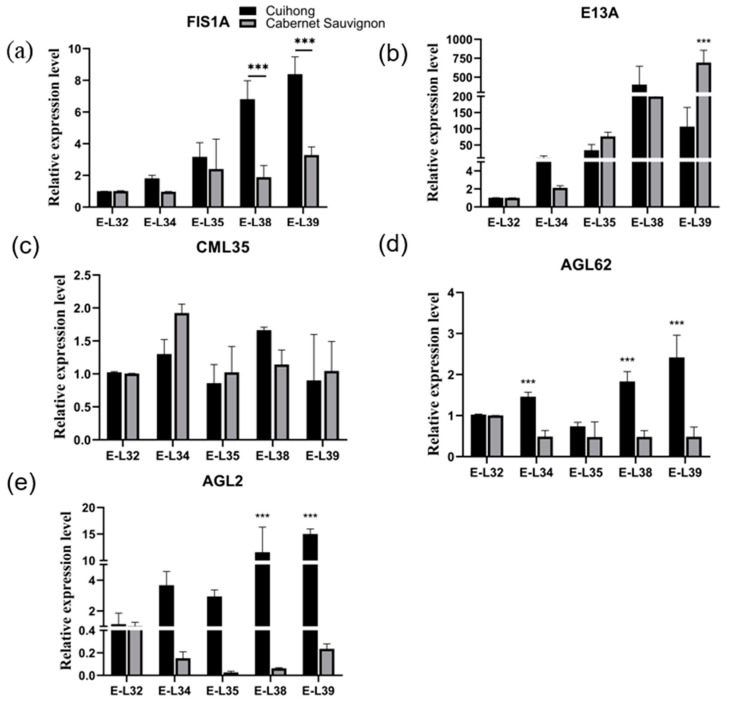
The expression levels of candidate genes at different growth stages (E-L 32, E-L 34, E-L35, E-L38, E-L39) of “Cuihong” and “Cabernet Sauvignon”. (**a**) *FIS1A*; (**b**) *E1A*; (**c**) *CML35*; (**d**) *AGL62*; (**e**) *AL2*. *** represents the significant difference between the control and experimental groups through analysis of variance.

The expression of candidate genes in these five periods was further verified by qRT-PCR. The expression of *E13A* was the highest in “Cabernet Sauvignon”’s over-ripe period, and reached the peak in “Cuihong”’s harvest-ripe period. During berry development, the expression of *E13A* was low in the first two periods, and increased rapidly in the last three periods, which was opposite to the change in sarcocarp firmness. Similarly, the expression of *FIS1A* in “Cabernet Sauvignon” and “Cuihong” showed a gradual increase trend, and reached the maximum at the harvest-ripe period of berries, the same trend as the decline in berry sarcocarp firmness. The gene expression of *CML5* in “Cabernet Sauvignon” was basically consistent with the change trend of pericarp hardness and peel toughness, showing a trend of rising and then slowly declining. The expression level of *AGL2* reaches its maximum during the over-ripening stage of “Cuihong” and the bunch closure stage of “Cabernet Sauvignon” (Figure 7).

The expression level of *AGL62* is highest during the ripening period of “Cuihong” and the softening stage of “Cabernet Sauvignon”. Due to the similar trend of expression levels of *E13A*, *CML35*, and *FIS1A* with changes in berry texture, it is speculated that these three genes may be related to berry texture. Therefore, these three genes were selected for subsequent transient expression experiments.

### 2.7. Transient Expression of Candidate Genes

**Figure 8:**
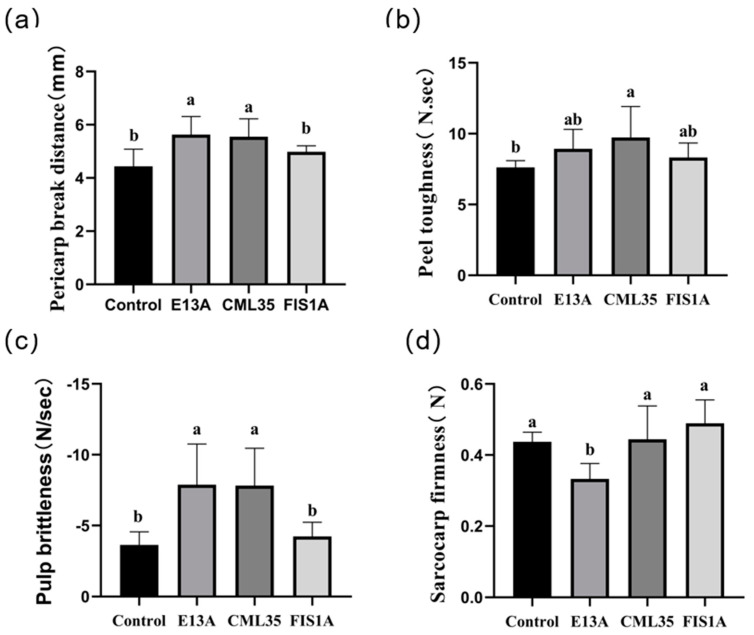
The effect of transient expression on berry texture. (**a**) Pericarp break distance; (**b**) Peel toughness; (**c**) Pulp brittleness; (**d**) Sarcocarp firmness. “a” and “b” represent significant differences, and when the letters are different, they indicate significant differences.

The genes *E13A*, *CML35*, and *FIS1A,* with similar relative expression levels during development and berry texture changes, were selected for transient transformation to preliminarily identify gene function (Supplementary Figures S4 and S5). The texture of the berries after 7 days of instantaneous transformation was measured, and there was no significant change in the peel hardness of these three genes after instantaneous transformation compared to the control group (Supplementary Figure S3). However, compared with the control group, the peel rupture distance and peel toughness of overexpressing *E13A* and *CML35* were significantly increased (Figure 8a,b). When the peel toughness increased, the displacement required to penetrate the peel also increased, indicating that the overexpression of *E13A* and *CML35* can increase the peel toughness, thereby increasing the cracking resistance of berries. In terms of texture, overexpression of *E13A* and *CML35* also significantly increased the brittleness of the pulp (Figure 8c). At the same time, after transient transformation of the *E13A* gene into fruit, the flesh firmness decreased significantly (Figure 8d), which was consistent with the results of qPCR, indicating that the expression level of the *E13A* gene increased with the decrease in flesh firmness. Unfortunately, *FIS1A* did not have a significant impact on the texture of the berries during this instantaneous transformation. 

The results of the instantaneous transformation indicate that *E13A* can increase the peel toughness, reduce the sarcocarp firmness, and have a significant impact on the overall texture of the berry. *CML35* has a relatively greater impact on the fruit peel, which is consistent with the previous qPCR results.

## 3. Discussion

Berry texture has a certain impact on fruit post-harvest processing, microbial safety, shelf life, consumer acceptability, and adaptability for further processing [28]. Meanwhile, studies have shown that maintaining good fruit texture can, to some extent, improve disease resistance and stress resistance during storage and transportation. The soft flesh of the grape will reduce the storage and commercial properties of the fruit, so fruit texture is very important for table grapes. Fruit softening is a sign of maturity of most fruits. Due to different species and cultivation conditions, the softening of fruit texture to a certain extent is acceptable, while excessive softening usually leads to the loss of flavor and marketability. A thicker pulp contributes to the elasticity of the fruit, enhancing its resistance to storage conditions and reducing the likelihood of cracking. This characteristic is highly desirable, as it not only preserves the integrity of the grape but also enriches its sensory experience [29]. Previous studies have shown that it is difficult to obtain high-sarcocarp firmness and high-pericarp hardness grape cultivars with high pericarp brittleness. At the same time, greater peel toughness and thick peel will also reduce the possibility of berry cracking [9].

In this study, we examined 437 grape accessions and observed that pericarp hardness, peel toughness, and sarcocarp firmness exhibited continuous variation and a normal distribution. These findings are consistent with the understanding that textural traits are quantitative characteristics of grapes [30,31,32]. Furthermore, the analysis of 437 grape accessions revealed that the majority of the five texture parameters exhibited either extremely significant or significant correlations. This finding aligns with previous research, underscoring that fruit texture is fundamentally governed by a multitude of interrelated parameters, reflecting an expression of complex, integrated traits [32]. Overall, the flesh of table grapes has a higher texture firmness, which is due to the emphasis on taste during the breeding process of table grapes. In the process of artificial selection and breeding, there is a preference for breeding directions with high firmness to meet consumers’ preferences for the texture of table grapes [33]. In contrast, wine grapes are more inclined towards characteristics such as juiciness and softness to adapt to the requirements for grape juice and flavor in the winemaking process [34]. Fruit softening is a natural indicator of ripeness across a variety of fruits. It is acknowledged that variations in species and cultivation conditions can lead to some degree of acceptable texture softening. However, excessive softening can result in a compromise of flavor and a diminished marketability [35]. The transformation of fruit texture is primarily driven by the decomposition of cell walls and alterations in the fruit cuticle structure. These changes are often the result of enzymatic actions within the cell walls, as well as the influence of other cell wall factors [36]. The cell wall, primarily composed of pectin, cellulose, and hemicellulose, undergoes significant changes during the ripening and softening of fruit. As cell wall enzymes act upon these components, they are degraded, leading to a relaxation of the wall’s structure and a consequent reduction in fruit hardness [37].

Many studies have also been conducted focusing on the texture traits of berries. Lin employed intraspecific hybridization between “Red Globe”, a cultivar with firm flesh, and “Muscat Hamburg”, characterized by its soft flesh, to establish an experimental population. Utilizing this population, a high-density genetic map was constructed [9]. Through QTL mapping, the gene encoding Glucan endo-1,3-β-glucosidase was pinpointed to linkage group 10 and identified as a promising candidate gene potentially associated with grape berry texture. In our study, chromosome 8 was located on *E13A* and annotated as glucan 1,3-β-glucosidase, which is involved in the metabolism of β-glucan. β-glucan is the main structural component of the cell wall. It can also function as a transglycosidase in biosynthesis. Through qPCR analysis, it was found that the expression level of E13A increases with the decrease in fruit hardness. At the same time, transient expression experiments also proved that this gene can reduce fruit hardness. This gene may promote cell wall degradation by regulating β-glucan, thereby reducing fruit hardness and promoting fruit softening.

Calcium plays an indispensable role in the growth and development of plants. As an integral component of the cell wall, it serves to connect adjacent cells and modulate enzyme activities. Its influence on fruit hardness is significant, as it mitigates the effects of cell wall-degrading enzymes, thereby potentially preserving the structural integrity of the fruit [38]. Balic discovered that table grape accessions with lower calcium content in their cell walls exhibit softer textures compared to those with higher calcium concentrations. In a related study [39], the authors identified dozens of genes within the QTL regions associated with fruit firmness in table grapes. Notably, one of these genes encoded a significant cation/calcium exchanger, highlighting its potential role in determining fruit texture. In this study, the *Vitvi08g01683* identified on chromosome 8 was annotated as encoding a possible calcium-binding protein CML35. The expression of this gene during the developmental stage is consistent with the trend of changes in the texture traits of the fruit peel. In transient expression, this gene increases the toughness of the fruit peel, which is consistent with the results of qRT-PCR. Therefore, it is speculated that this gene may be an important candidate gene affecting the essence of pulp fruit. Guo et al. genotyped 179 grape accessions using simplified genome sequencing techniques and performed a genome-wide association analysis focusing on the texture of grapes [40]. This analysis led to the identification of two SNP-associated genes on chromosome 16 that are implicated in calcium regulation, further underscoring the role of calcium in berry texture. 

The MADS-box family of genes encodes transcription factors that play critical roles in various plant development processes, particularly fruit development and maturation. Overexpression of the MADS-box gene *SlMBP3* in tomatoes leads to an abnormally soft phenotype in fruits [41]. Daminato downregulated the MADS-box gene *FaSHP* in strawberries and observed significant inhibition of three genes related to the fruit softening process [42]. In this study, MADS-box transcription factors *AGL2* and *AGL62* were identified through gene annotation. In order to further investigate their potential roles, qRT-PCR was used to evaluate the expression of these two genes at different developmental stages and the trend of fruit texture changes. It was found that there was no significant correlation, and their functions may be exerted through synergistic effects with other genes.

*FIS1* is a key catabolic enzyme that affects the levels of active GA in tomato fruits. It regulates the expression of genes related to the synthesis of the stratum corneum, thereby affecting the thickness and composition of the stratum corneum. Therefore, *FIS1* plays an important role in determining the hardness of tomatoes. Li used CRISPR editing technology to generate FIS1cr mutants in tomatoes, demonstrating that it can effectively increase tomato hardness without affecting fruit quality [43]. Our study identified the vitvi015368 gene on chromosome 8, annotated as *FIS1A*. The expression of this gene is consistent with the decreasing trend of fruit flesh hardness; therefore, transient expression is performed, but unfortunately, this gene does not have a significant effect on the texture of berries.

During the transient expression process, when the concentration of the inoculation solution is too low, the number of Agrobacterium is insufficient, which will reduce the rate of transient expression; when the density of Agrobacterium is too high, the intense competition among Agrobacterium is not conducive to the binding of the bacteria with plant cells, thereby affecting the rate of gene transient expression [44]. Transient expression vectors are used to introduce foreign genes into host cells, allowing the target genes to be highly expressed or silenced in a short period of time. However, the free foreign DNA does not integrate into the host cell’s chromosomal DNA, and therefore cannot be stably inherited [45]. Transient expression results vary in different trials and different plants, making it difficult to implement on a large scale in horticultural plants, with poor repeatability. The results obtained from transient expression in this experiment can serve as a preliminary verification direction for later stages, and the gene function can be verified in the future through stable genetic transformation in tomatoes. 

In conclusion, this study evaluated the texture characteristics of various grape accessions using the puncture method. A comprehensive genome-wide association analysis was conducted by combining phenotype data of berry texture with the results of our previous population genome sequencing. Through GWAS, expression analysis, and transient expression, key candidate genes *E13A* and *CML35* related to grape berry texture were identified. This finding provided valuable insights for improving grape texture and laid the foundation for the development of hard flesh grape accession in breeding programs.

## 4. Materials and Methods

### 4.1. Experimental Materials

All plant materials were harvested from 437 grape accessions at the Zhengzhou Fruit Research Institute of the Chinese Academy of Agricultural Sciences (113°42′ E and 34°48′ N, an altitude of 110.4 m). The nursery benefits from an annual average temperature of 14.2 °C, annual rainfall of 666.0 mm, 2436 h of sunshine, and a 213-day frost-free period. The soil is characterized as brown with a sandy loam texture, exhibiting a slightly alkaline pH ranging from 7.1 to 7.5. The grapevines are cultivated using a single-trunk double-arm trellis system, set at a height of 1.8 m with a plant and row spacing of 1.0 m by 2.5 m, oriented in a north–south direction, and maintained at a medium level of nursery management.

### 4.2. Experimental Method

#### 4.2.1. Experimental Material Collection

From July to October 2023, grape harvesting was carried out every morning from 7 to 9 o’clock. Based on the development status of the berries and the degree of seed browning, samples were taken (the seeds were dark brown and the soluble solids content generally reached 18%) to ensure the measurement of the texture of mature berries.

Each accession was repeated for each tree, with a total of 3 replicates. A total of 10 berries were randomly selected for testing. After the berries were put in the marked self-sealed bag, they were quickly returned to the laboratory and tested within 1 day. Before the experiment, the berries should be screened, the diseased and rotten fruits should be removed, and the berries with basically the same maturity and size should be selected.

#### 4.2.2. Measurement of Grape Berry Texture

The grape texture was assessed using a modified whole fruit puncture test method [9]. The testing was conducted with a TA XT plus texture analyzer, employing a 2 mm diameter P/2 probe. The settings for the test parameters were as follows: pre-test speed at 1 mm/s, penetration speed at 1 mm/s, post-test speed at 10 mm/s, puncture depth set to 7 mm, and the load trigger force at 5 g. During the test, place the grape berry with short fruit stalk on the loading platform of the texture analyzer, fix it slightly by hand, but do not press the fruit. The probe should not touch the seed during puncture, so as not to affect the test results.

The related indices include pericarp hardness; pericarp break distance; peel toughness; pulp brittleness; sarcocarp firmness.

#### 4.2.3. Genome-Wide Association Studies

The VCF file was filtered by PLINK2 (parameters: 0-maf 0.05, -hwe 1 × 10^−5^ and -geno 0) to obtain high-quality SNP markers. Using GEMMA 7B, the obtained SNPs and the five grapevine surface type traits determined in this experiment were analyzed by using the Wald test through the linear mixed model (−1 mm). A Manhattan map was drawn using R language, and candidate genes were extracted using Python Software Foundation (2019) within ± 5 KB of significant SNPs. In this GWAS analysis, we used a linear regression model to identify associations between genetic variants and continuous traits. To control for population structure and relatedness, we applied a mixed linear model. To correct for Type I errors arising from multiple testing, we used the Wald test to obtain *p* values, followed by a Bonferroni correction (y = −log10[0.05 per number of variants]) as the significance threshold to control for false positive rates (Type I error).

#### 4.2.4. Measurement of Grape Texture and qRT-PCR During the Development Stage

At five distinct developmental stages, namely E-L 32 (bunch closure stage), E-L 34 (softening stage), E-L 35 (veraison stage), E-L 38 (ripening stage), and E-L 39 (over-ripening stage), Cabernet Sauvignon and “Cuihong” were harvested. Approximately 20 berries were randomly harvested from three individual grapevines. A portion of these berries was utilized for the assessment of berry texture, with the documentation of fundamental parameters such as individual berry weight and total soluble solids content, along with photographic records to capture the morphological characteristics at each stage. 

The remaining berries were immediately cryopreserved in liquid nitrogen and then stored at −80 ° C for subsequent RNA extraction. Total RNA was extracted from the fruit flesh and reverse-transcribed into complementary DNA (cDNA). Quantitative real-time polymerase chain reaction (qRT-PCR) was conducted using the Roche 480 Light Cycler system (Roche Diagnostics, Indianapolis, IN, USA), with the housekeeping gene GAPDH serving as an internal control. Each candidate gene was subjected to three biological replicates, and the relative expression levels of the genes were determined using the 2^−∆∆Ct^ method. qRT-PCR primers are listed in Table S4.

#### 4.2.5. Transient Expression of Candidate Genes in Grapes

Using grape leaf cDNA as a template, gene cloning was performed based on the CDS sequences of E13A, CML35, and FIS1A downloaded from the Ensembl Plants database. Plasmids were extracted using the plasmid extraction kit (DP103, TIANGEN, BeiJing). By homologous recombination, the CDS sequence of the gene was inserted into a pSAK277 linear vector containing XBAI and HindIII HF sites to generate an overexpression recombinant vector. Transform pSAK277-E13A, pSAK277-CML35, pSAK277-FIS1A, and empty pSAK277 vectors into Agrobacterium tumefaciens strain GV3101. After shaking at 28 °C for 16 h, adjust the OD600 to approximately 0.8 using resuspended solution (10 mM MES, 10 mM MgCl_2_, and 100 μM acetosyringone) to achieve optimal infection status. Select uncolored “Cabernet Sauvignon”, cut off the berries, and steep under running water for 12–16 h. Pour the infection solution into the boxes separately, place the pre-processed berries on the holes, and ensure that the fruit stems are soaked in the infection solution. Cultivate for 7 days under the conditions of 28 °C and 16 h/8 h photoperiod [46].

### 4.3. Statistical Analysis 

Both statistical and genome-wide association studies (GWAS) were conducted using the mean values of various traits. Phenotypic data for five texture traits are presented in Table S1 and were analyzed using Microsoft Excel 2013 (Microsoft Corporation, Redmond, WA, USA) and SPSS 19.0 for Windows (SPSS Inc., Chicago, IL, USA). The texture of the grapes was subjected to cluster analysis using R 4.3.2, while the correlation among traits was examined with the Corrplot tool in Origin 8.0 software (OriginLab, Northampton, MA, USA).

## Figures and Tables

**Table 1:** Parameters of grape texture.

Texture Parameters	Mean	Max	Min	SD	CV
PPH (N)	3.488	9.089	1.173	1.195	0.342
PBD (mm)	3.496	6.415	0.031	1.448	0.414
PT (N.s)	6.982	19.813	0.859	3.584	0.513
PB (N/s)	−34.567	−0.482	−196.747	38.686	−1.119
SF (N)	0.476	1.843	0.091	0.225	0.471

**Table 2:** Selected candidate gene information related to grape berry texture.

SNP ID	*p* Value	Gene ID	Gene Name	Annotation
11_3126218	6.88 × 10^−7^	Vitvi11g00329	*MPV17*	Protein Mpv17
15_15166809	3.27 × 10^−8^	Vitvi15g00563	*NRG2*	Nitrate regulatory gene2 protein
15_15166809	3.27 × 10^−8^	Vitvi15g00564	*LCMT1*	Leucine carboxyl methyltransferase 1 homolog
8_21082265	1.33 × 10^−6^	Vitvi08g01701	*E13A*	Glucan 1,3-beta-glucosidase
8_20379794	2.78 × 10^−8^	Vitvi08g01645	*SMG7*	Nonsense-mediated mRNA decay factor SMG7
8_20671485	1.58 × 10^−6^	Vitvi08g02313	*RPE5A*	DNA-directed RNA polymerase V subunit 5A
8_20671485	1.58 × 10^−6^	Vitvi08g02314	*FIS1A*	Mitochondrial fission 1 protein A
8_20250484	4.41 × 10^−6^	Vitvi08g01968	*AGL2*	Alpha-glucosidase 2
8_21018177	4.08 × 10^−6^	Vitvi18g01501	*AGL62*	Agamous-like MADS-box protein AGL62
18_22038237	5.68 × 10^−7^	Vitvi18g01579	*Y4967*	Uncharacterized oxidoreductase At4g09670
8_11881442	7.17 × 10^−6^	Vitvi08g01683	*NLTP5*	Non-specific lipid-transfer protein P5
8_20916540	8.51 × 10^−6^	Vitvi08g01683	*CML35*	Probable calcium-binding protein CML35

## Data Availability

The original contributions presented in this study are included in the Supplementary Materials. Further inquiries can be directed to the corresponding authors.

## Supplementary Materials

The following supporting information can be downloaded at: https://www.mdpi.com/article/10.3390/ijms252313065/s1.
