# Supplementary material for: Genome-Wide Association Study of Grape Texture Based on Puncture
Source: Int J Mol Sci. 2024 Dec 5;25(23):13065. doi: 10.3390/ijms252313065 (PMC11642401; doi:10.3390/ijms252313065)
Supplement: Supplementary file 1 [file ijms-25-13065-s001.zip › supplementary figure.pdf]

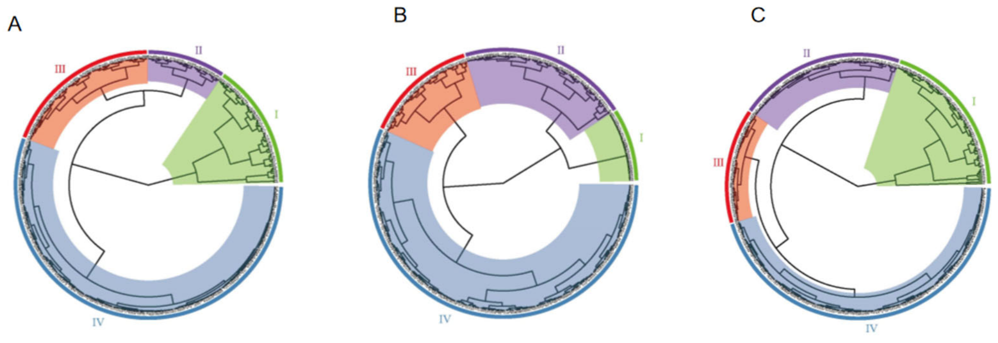

supplementary figure S1. Cluster analysis of texture traits of 437 grape germplasms. A:Pericarp hardness; B: Peel toughness; C: Sarcocarp firmness.

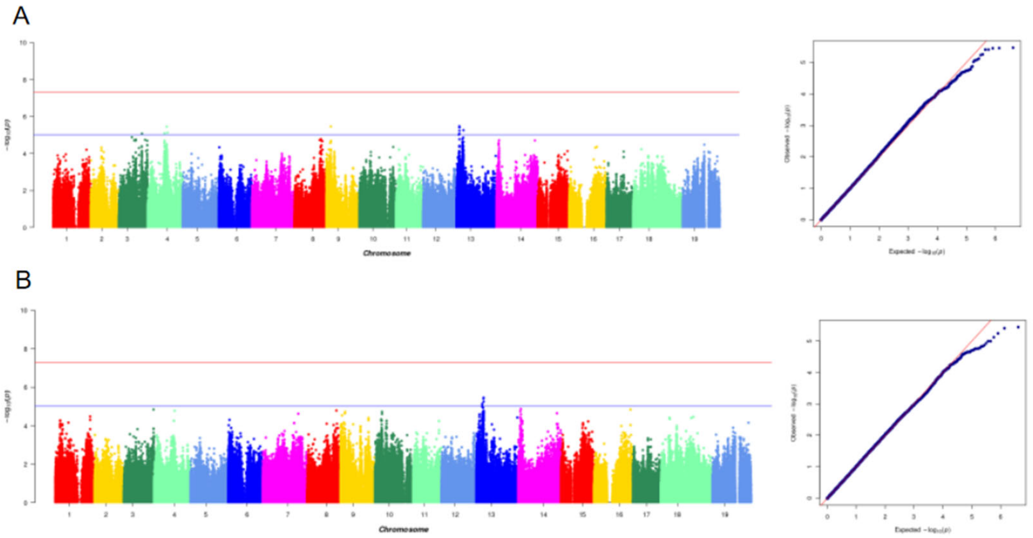

supplementary figure S2. Genome-wide association analysis (GWAS) Manhattan plot and Q-Q plot of A:Pericarp hardness; B: Peel toughness.

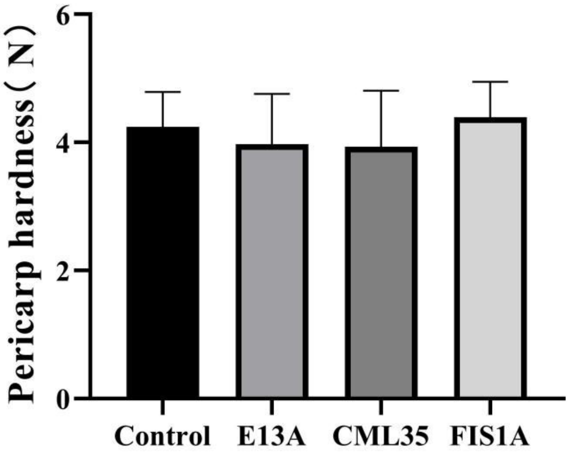

supplementary figure S3. The Effect of Transient Expression on Pericarp hardness

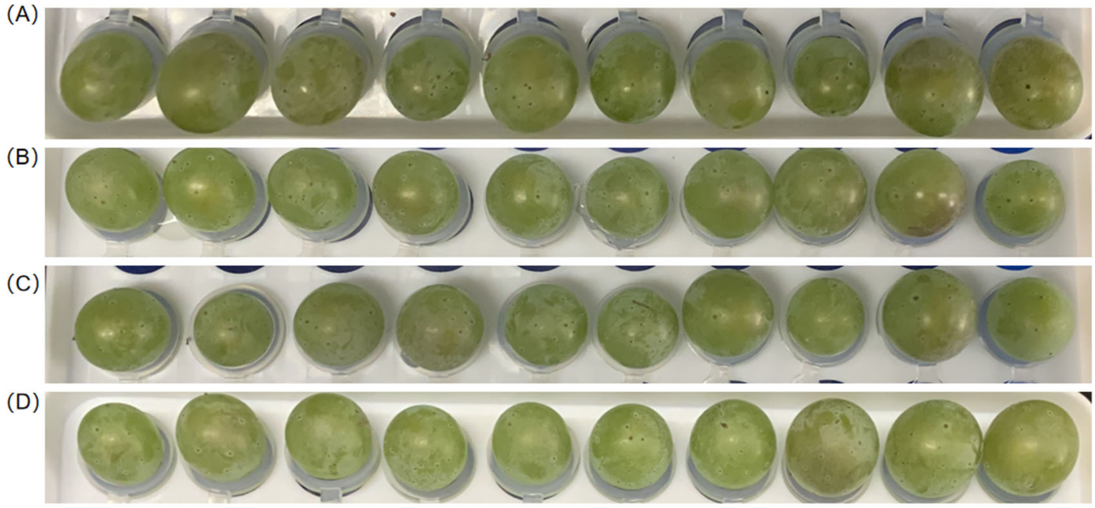

supplementary figure S4. Berries before transient expression.(A)CML35;(B)E13A;(C)FIS1A(D)Control

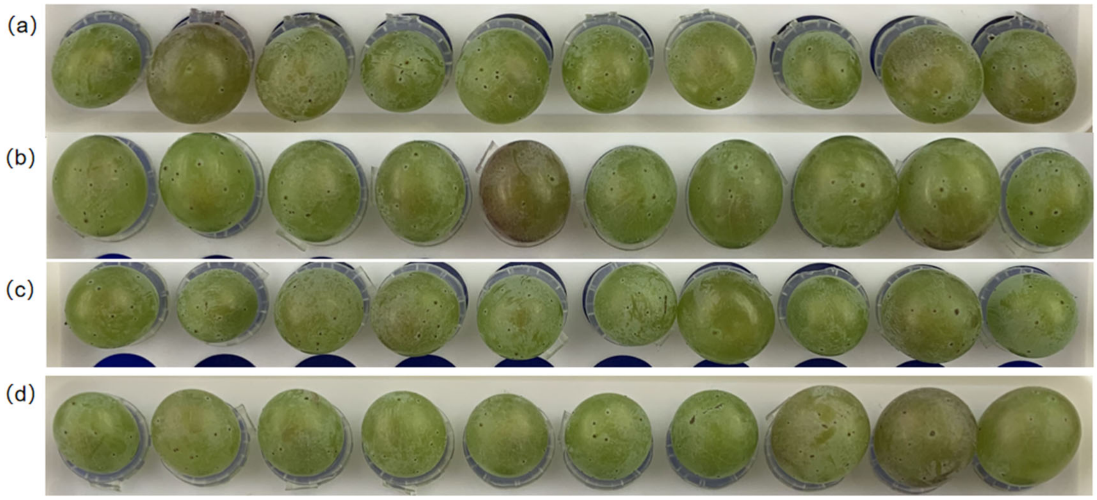

supplementary figure S5. Berries after transient expression.(a)CML35;(b)E13A;(c)FIS1A(d)Control
